# Supplementary material for: Hilbert space fragmentation at the origin of disorder-free localization in the lattice Schwinger model
Source: Commun Phys. 2025 Apr 18;8(1):172. doi: 10.1038/s42005-025-02039-8 (PMC12008026; doi:10.1038/s42005-025-02039-8)
Supplement: Supplementary file 1 — Supplementary Information [file 42005_2025_2039_MOESM1_ESM.pdf]

Supplementary Information for "Hilbert space fragmentation at the origin of disorder-free localization in the lattice Schwinger model"

CONTENTS

Supplementary Note 1: Derivation of the lattice gauge theory — i

Supplementary Note 2: Jordan-Wigner mapping — ii

Supplementary Note 3: Other initial states — ii

Supplementary Note 4: Derivation of the resonance condition — iii

Supplementary Note 5: Comparison with disordered XXZ model — iv

Supplementary Note 6: Spectral form factor — v

Supplementary Note 7: Many-body Thouless parameter — vi

Supplementary Note 8: Coulomb interactions versus disorder — vi

Supplementary Methods: Degenerate perturbation theory for the lattice Schwinger model — vii

Supplementary References — vii

SUPPLEMENTARY NOTE 1: DERIVATION OF THE LATTICE GAUGE THEORY

In this section, we revisit the derivation of the lattice Schwinger model. As a model of Quantum Electrodynamics (QED), the Schwinger model describes interactions between spinless fermions and antifermions via electric fields in one spatial dimension. In this case, the vector potential $\hat{A}(x)$ has a temporal and a spatial component given by $(\hat{A}_0(x), \hat{A}_1(x))$. We fix a gauge by setting $\hat{A}_0(x) = 0$. In this gauge, the electric field operator becomes $\hat{E}(x) = -\partial_0 \hat{A}_1(x)$ where ∂_0 is the partial time derivative. The electric field operator is the canonical momentum conjugate to the vector potential $\hat{A}(x)$ with the commutation relation $[\hat{A}_1(x), \hat{E}(x')] = -i\delta(x - x')$. The model consists of a matter field $\hat{\Phi}(x)$ which is a two-component spinor field $(\hat{\Phi}_{e^-}(x), \hat{\Phi}^\dagger_{e^+}(x))^T$ representing electrons and positrons.

In the continuum limit with natural units $\hbar = c = 1$, the Schwinger model has a $U(1)$ gauge symmetry and the Hamiltonian is given by:

$$\hat{H}_{\text{cont}} = \int dx \left(-i\bar{\Phi}(x)\gamma^1 D_1 \hat{\Phi}(x) + m\bar{\Phi}(x)\hat{\Phi}(x) + \frac{1}{2}\hat{E}^2(x) \right) , \quad \text{(S1)}$$

where $D_1 = \partial_1 - ig\hat{A}_1(x)$ is the gauge covariant derivative in one spatial dimension, ∂_1 is the partial derivative with respect to x, and m is the fermionic mass. The Dirac adjoint of the spinor field is defined as $\bar{\Phi} = \hat{\Phi}^\dagger\gamma^0$, where $(\gamma^0, \gamma^1) = (\hat{\sigma}^z, i\hat{\sigma}^y)$ are the Dirac matrices in 1+1-dimension. We set the coupling constant $g = e$ as the charge e of the electrons.

Moving away from the continuum, this model can also be formulated on a one dimensional lattice where points in space are separated by a distance a and time is continuous. In particular, we use the lattice version first developed by Kogut and Susskind [1, 2]. In this formulation, the two-component matter field $(\hat{\Psi}_{e^-}(x), \hat{\Psi}^\dagger_{e^+}(x))^T$ is 'unfolded' onto the lattice, by placing the electron/positron fields onto alternating even/odd sites, with two neighboring sites defining a unit cell. The discrete versions of the vector potential and the electric fields are placed on the links connecting neighbouring lattice sites.

To respect the $U(1)$ symmetry of the original model, we move from the canonically conjugate continuous fields $\hat{E}(x)$, $\hat{A}_1(x)$ to their corresponding discrete versions $\hat{\phi}_n$, $\hat{L}_n$ such that the canonical commutation relations are

$$[\hat{A}_1(x), \hat{E}(x')] = -i\delta(x - x'), \quad [\hat{\phi}_n, \hat{L}_m] = i\delta_{n,m}, \quad \text{(S2)}$$

where the $\hat{\phi}_n = ag\hat{A}_1(x_n)$ is a $U(1)$ parallel transporter and $\hat{L}_n = (-1/g)\hat{E}(x_n)$ is the canonically conjugate electric field defined on the link between neighboring sites $(n, n+1)$. The vector potential $\hat{A}_1(x_n)$ enters the Hamiltonian through an exponential term of the form $e^{i\hat{\phi}_n} = e^{-iag\hat{A}_1(x_n)}$, which requires $0 \leq \phi(n) \leq 2\pi$, where $\phi(n)$ represents the eigenvalue of $\hat{\phi}_n$. Looking at the commutation relation between $\hat{\phi}_n$ and $\hat{L}_n$, it is straightforward to see that $\hat{L}_n$ generates cyclic translations in $\hat{\phi}_n$ and is thus an angular momentum operator. As a consequence of the range of $\hat{\phi}_n$ and Eq. (S2), $\hat{L}_m$ is quantized with integer eigenvalues,

$$\hat{L}\,|L\rangle = \hat{L}\,|L\rangle\,, \quad \hat{L} = 0, \pm1, \pm2... \quad \text{(S3)}$$

Then, $e^{i\hat{\phi}_n}$ acts as a ladder operator on the eigenstates $|l\rangle$ of $\hat{L}_m$ as:

$$e^{\pm i\hat{\phi}}\,|l\rangle = \pm\,|l \pm 1\rangle\,. \quad \text{(S4)}$$

We define one component fermionic field operators on each site as

$$\hat{\Psi}_n = \begin{cases} \sqrt{a}\hat{\Phi}_{e^-}(x_n) & \text{for even } n \\ \sqrt{a}\hat{\Phi}^\dagger_{e^+}(x_n) & \text{for odd } n \ . \end{cases} \tag{S5}$$

We can now write down the lattice Schwinger Hamiltonian on a chain of N sites with open boundary conditions by replacing the integrals in Eq. (S1) with discrete sums as

$$\begin{aligned} \hat{H}_{\text{sch}} &= -iw \sum_{n=1}^{N-1} [\hat{\Psi}_n^\dagger \hat{U}_n \hat{\Psi}_{n+1} - \text{H.c.}] \\ &+ J \sum_{n=1}^{N-1} \left(\hat{L}_n + \frac{\theta}{2\pi} \right)^2 + m \sum_{n=1}^{N} (-1)^n \hat{\Psi}_n^\dagger \hat{\Psi}_n \ , \end{aligned} \tag{S6}$$

with $\hat{U}_n = e^{i\hat{\phi}_n}$. The first term in (S6) describes nearest-neighbor hopping and corresponds to the creation and annihilation of electron-positron pairs. The strength of this interaction is given by $w = 1/2a$. The second term describes the rest mass energy (proportional to the fermionic mass m), while the last term describes the energy stored in the electric fields with $J = g^2a/2$. The lattice Hamiltonian reduces to the original Schwinger Hamiltonian in Eq. (S1) in the limit $a \to 0$. We have further included a constant background field $\theta/2\pi$, also known as the topological θ term.

States in the Hilbert space are constrained by Gauss' law, which takes the form $\partial_1 \hat{E}(x) = g\bar{\Phi}\gamma^0\hat{\Phi}$ in the continuum limit. In the lattice version, Gauss' law can be formulated by considering a set of on-site generators

$$\hat{G}_n = \hat{L}_n - \hat{L}_{n-1} - \hat{\Psi}_n^\dagger \hat{\Psi}_n + \frac{1}{2}[1 - (-1)^n] \tag{S7}$$

such that $[\hat{H}_{\text{sch}}, \hat{G}_n] = 0$. *Physical* states in the Hilbert space are defined as eigenvectors of $\hat{G}_n$, such that,

$$\hat{G}_n \left|\psi_{\text{phys.}}\right\rangle = q_n \left|\psi_{\text{phys.}}\right\rangle \ , \tag{S8}$$

where q_n represents the distribution of background charges. The fact that $\hat{G}_n$ commutes with the Hamiltonian has an important consequence on the dynamics of the system: $\hat{H}_{\text{sch}}$ does not mix states with different q_n during dynamics, thus $\hat{H}_{\text{sch}}$ is block-diagonal is the eigenbasis of $\hat{G}_n$. The q_n's represent static background charges in the theory that act as sources of electric flux.

SUPPLEMENTARY NOTE 2: JORDAN-WIGNER MAPPING

We may recast the lattice Schwinger Hamiltonian from Eq. (S6) as a spin model by a Jordan-Wigner transformation of the fermionic fields. Writing $\hat{\Phi}_n = \prod_{l<n}[i\hat{\sigma}_l^z]\hat{\sigma}_n^-$ ($\hat{\Phi}_n^\dagger = \prod_{l<n}[-i\hat{\sigma}_l^z]\hat{\sigma}_n^+$) and using a gauge transformation $\hat{\sigma}_n^\pm \Rightarrow \prod_{l<n}[e^{\mp i\hat{\phi}_l}]\hat{\sigma}_n^\pm$, we arrive at the spin Hamiltonian

$$\begin{aligned} \hat{H}_{\text{spin}} &= \hat{H}_\pm + \frac{m}{2}\sum_{n=1}^{N}(-1)^n\hat{\sigma}_n^z + J\sum_{n=1}^{N-1}\hat{L}_n^2, \\ \hat{H}_\pm &= w\sum_{n=1}^{N-1}(\hat{\sigma}_n^+\hat{\sigma}_{n+1}^- + \text{H.c.}). \end{aligned} \tag{S9}$$

Now, we integrate out the gauge fields using Gauss' law. In the spin language, the Gauss' law constraint becomes $\hat{L}_n - \hat{L}_{n-1} = \frac{1}{2}[\sigma_n^z + (-1)^n] + q_n$. By setting the boundary fields $\hat{L}_0 = \hat{L}_N = 0$, we get

$$\begin{aligned} &\sum_{n=1}^{N-1}\left(\hat{L}_n + \frac{\theta}{2\pi}\right)^2 \\ &= \sum_{n=1}^{N-1}\left(\sum_{m=1}^{n}\left[\frac{1}{2}[\hat{\sigma}_m^z + (-1)^m] + q_m\right] + \frac{\theta}{2\pi}\right)^2 \ , \end{aligned} \tag{S10}$$

where we have now included the topological background field θ. Representing the energy stored in the electric fields, this term can be separated into two parts consisting of a long-ranged two-body term $\hat{H}_{ZZ}$, and a field term $\hat{H}_q$ dependent on the charge configuration:

$$\begin{aligned} \hat{H}_{ZZ} &= \frac{J}{2}\sum_{j=1}^{N-2}\sum_{k=j+1}^{N-1}(N-k)\hat{\sigma}_j^z\hat{\sigma}_k^z \ , \\ \hat{H}_q &= \frac{m}{2}\sum_{j=1}^{N}(-1)^j\hat{\sigma}_j^z \\ &+ \frac{J}{2}\sum_{k=1}^{N-1}\sum_{j=k}^{N-1}\left[2\left(\sum_{i=1}^{j}q_i\right) - (j \mod 2) + \frac{\theta}{\pi}\right]\hat{\sigma}_k^z \ . \end{aligned} \tag{S11}$$

Curiously, the long-range term $\hat{H}_{ZZ}$ which represents Coulomb interactions and the term contributing to on-site disorder $\hat{H}_q$ both originate from the same term in the original Hamiltonian. Unlike the conventionally studied MBL models such as the random field XXZ chain where interactions and disorder can be tuned independently, a single parameter J controls both the interactions and the strength of disorder in this model.

SUPPLEMENTARY NOTE 3: OTHER INITIAL STATES

In the main text, when we refer to thermalization or ergodicity breaking, we have in mind the generic behavior of the system from typical initial states. However, both our main text and previous literature [3] have focused on the dynamical properties of the Schwinger model prepared in a single $|\text{vac}\rangle$ initial state. How typical is the $|\text{vac}\rangle$ initial state? Although $|\text{vac}\rangle$ is the bare vacuum of

staggered fermions, it is not the ground state of the spin model, with its energy given by

$$E_{\mathrm{vac}} = -\frac{J}{2}\left(\frac{N^2}{4} - \sum_{k=1}^{N-1}\left\lceil\frac{N-k}{2}\right\rceil q_k\right) . \quad \text{(S12)}$$

For generic background charges q_k's, we find that E_{vac} is close to but smaller than the energy at the DOS peak and so we expect its dynamical behavior to be representative of a typical initial state. In Supplementary Figure 1(a) and (b), we plot the growth of the charge sector averaged entanglement entropy S_E from all initial product states in the computational basis for $J/w = 1$, wherein we average over 150 charge sectors for each initial state. Although most initial states show a quick growth and saturation in number entropy, we observe qualitatively different behavior in the growth of configurational entropy. We find that $\sim 70\%$ of initial states show a relatively quick growth and saturation to steady-state values ($S_C > 0.9$), whereas the remaining initial states show a slower growth of entanglement over large timescales. The $|\mathrm{vac}\rangle$ state behaves typically and lies in the former group of states with a faster growth of S_C. Although the variability between initial states increases upon increasing J/w, the growth of entanglement from the $|\mathrm{vac}\rangle$ state remains qualitatively similar to the majority of initial states. We note that at large-J, fragmentation occurs at the level of the Hilbert space, thus affecting the dynamics of all initial states.

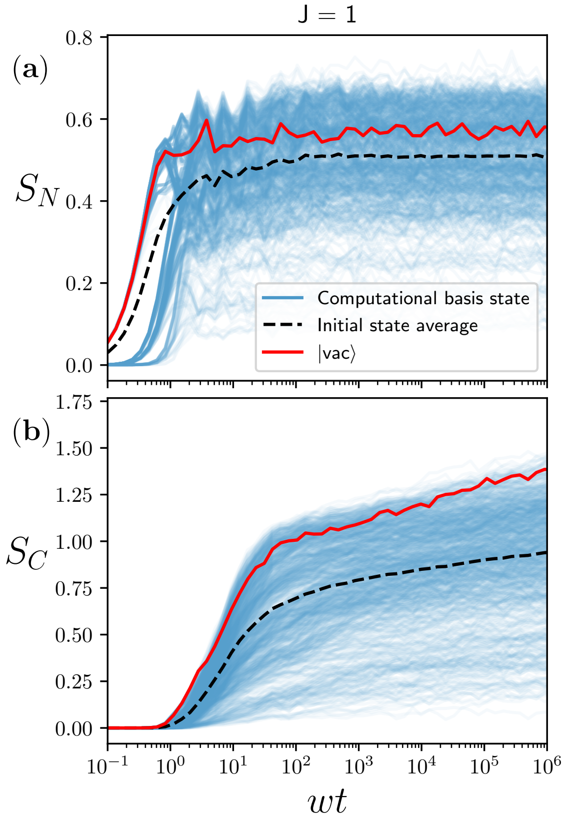

Supplementary Figure 1. Growth of (a) number and (b) configurational entropy from all initial computational basis states for $J = 1$ and $N = 12$. The entropy series for each initial state is averaged over 150 charge sectors.

SUPPLEMENTARY NOTE 4: DERIVATION OF THE RESONANCE CONDITION

Let us examine the energy shift when we interchange two spins at sites ℓ and $\ell+1$, $|01\rangle \to |10\rangle$, under the action of the $\hat{H}_\pm$ term in Eq. (S9). First, noting that $\hat{\sigma}^z_\ell\hat{\sigma}^z_{\ell+1}$ stays constant, let us explicitly write out all terms in $\hat{H}_{ZZ}$ which contribute to the shift:

$$\Delta\hat{H}_{ZZ} = \frac{J}{2}\left(\sum_{j=1}^{\ell-1}(N-\ell)\hat{\sigma}^z_j\hat{\sigma}^z_\ell + \sum_{j=1}^{\ell-1}(N-\ell-1)\hat{\sigma}^z_j\hat{\sigma}^z_{\ell+1} + \sum_{k=\ell+2}^{N-1}(N-k)\hat{\sigma}^z_\ell\hat{\sigma}^z_k + \sum_{k=\ell+2}^{N-1}(N-k)\hat{\sigma}^z_{\ell+1}\hat{\sigma}^z_k\right) . \quad \text{(S13)}$$

We can see that the second line is invariant under $\hat{\sigma}^z_\ell \leftrightarrow \hat{\sigma}^z_{\ell+1}$, and so we can reduce to the first line. Factorizing this, we obtain

$$\begin{aligned}\Delta\hat{H}_{ZZ} &= \frac{J}{2}\left[(N-\ell)\hat{\sigma}^z_\ell + (N-\ell-1)\hat{\sigma}^z_{\ell+1}\right]\sum_{j=1}^{\ell-1}\hat{\sigma}^z_j \\ &= \frac{J}{2}\hat{\sigma}^z_\ell\sum_{j=1}^{\ell-1}\hat{\sigma}^z_j ,\end{aligned} \quad \text{(S14)}$$

where we have used the fact that $\hat{\sigma}^z_\ell + \hat{\sigma}^z_{\ell+1} = 0$. Since $\hat{\sigma}^z_\ell$ goes from -1 to $+1$, the energy change of this hop due to $\hat{H}_{ZZ}$ is then $J\sum_{j=1}^{\ell-1}\hat{\sigma}^z_j$. At the same time, $\hat{H}_q$ changes by $2(m + h_\ell - h_{\ell+1})$. Therefore, substituting in the expression for h_k, we obtain the condition for resonant hopping with $m = 0$,

$$\frac{\Delta E}{J} \equiv \sum_{j=1}^{\ell-1}\hat{\sigma}^z_j + 2\sum_{j=1}^{\ell} q_j + \frac{\theta}{\pi} - (\ell \mod 2) = 0 . \quad \text{(S15)}$$

We may also see that, when the resonance condition is not met and $\theta/\pi = \pm 1$, the energy always changes in increments of $2J$. This follows since $J\sum_{j=1}^{\ell-1}\hat{\sigma}^z_j - (\ell \mod 2)$ is always odd, while $2\sum_{j=1}^{\ell} q_j$ is evidently even, and so the LHS of Eq. (S15) is even if θ/π is odd. For all other values of θ, the resonance condition can never be satisfied, and in particular for $\theta/\pi = 0$, ΔE is at least J.

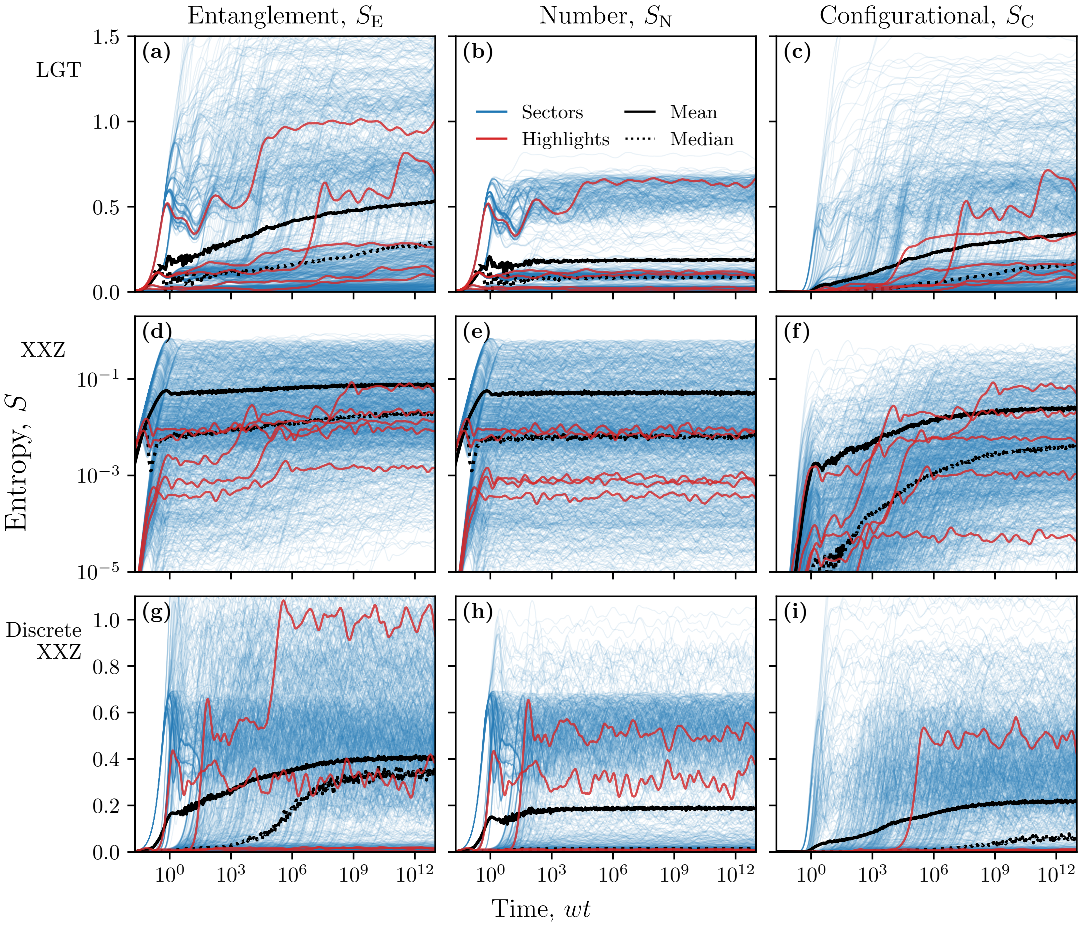

Supplementary Figure 2. (a-c) The entanglement entropy S_E, number entropy S_N, and configurational entropy S_C, respectively, for the lattice Schwinger model with $J = 5$, following a quench from $|\text{vac}\rangle$. Results for 1000 charge sectors are shown in blue; five randomly-chosen trajectories have been highlighted in red to help guide the eye. We also show the charge sector mean (black solid line) and median (black dashed line). (d-f) The same, but for the uniform-disorder XXZ model (S16) with $W = 20$. (g-i) The same, but for the discrete-disorder XXZ model with $W_j \in \{-W, 0, +W\}$ and $W = 20$.

SUPPLEMENTARY NOTE 5: COMPARISON WITH DISORDERED XXZ MODEL

In Supplementary Figure 2, we compare the behavior of the lattice Schwinger model with that of the disordered XXZ model, the prototypical model of MBL:

$$\hat{H}_{\text{XXZ}} = \sum_{j=1}^{N-1} J_{XY} \left(\hat{\sigma}_j^+ \hat{\sigma}_{j+1}^- + \hat{\sigma}_j^- \hat{\sigma}_{j+1}^+ \right) + \sum_{j=1}^{N-1} \frac{J_Z}{2} \hat{\sigma}_j^z \hat{\sigma}_{j+1}^z + \sum_{j=1}^{N} W_j \hat{\sigma}_j^z \,, \tag{S16}$$

where W_j is the quenched disorder potential drawn uniformly from the interval $[-W, W]$. Panels (a), (b) and (c) of Supplementary Figure 2 show the entanglement entropy S_E, number entropy S_N, and configurational entropy S_C, respectively, for the Schwinger model with $J = 5$. We observe the characteristic slow growth in entanglement entropy, dominated at late times by the configurational entropy. We also observe the "jump" behavior in the configurational entropy, and banding in all three plots.

These can be compared to Supplementary Figure 2(e-f), which depict the same for the XXZ model with $J_{XY} = J_Z = 1$ and $W = 20$. Here, we see that the entanglement entropy very quickly saturates, and that the number entropy is dominant at all times. The configurational entropy S_C appears to exhibit some sort of slow growth, with a much smaller saturation value than S_N, but jumps are less prominent, and no banding is visible in any of the three plots. It is clear that the phenomenology of entropy growth in the Schwinger model is distinct from that of "typical" random uniform disorder MBL.

However, there is a possibility that the observed behavior results from the discrete nature of the disorder. To that end, we also look at a version of the XXZ model where W_j instead takes on the discrete values

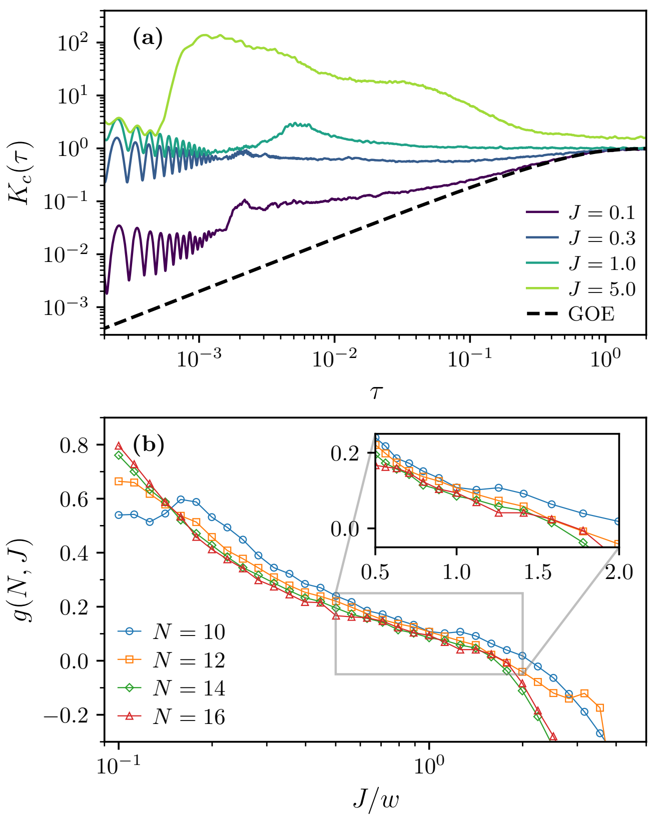

Supplementary Figure 3. (a) The connected spectral form factor (SFF), $K_c(\tau)$, for selected J at $N = 16$ (and $w = 1$). We also show the GOE ensemble value as the black dashed line. (b) $g(N, J) = \log_{10}(\tau_{\mathrm{H}}/\tau_{\mathrm{GOE}})$, which measures when the SFF converges to the GOE value, for various N as a function of J/w. In the thermodynamic limit, we expect $g \to +\infty$ ($g \to -\infty$) for chaotic (non-ergodic) systems.

$\{-W, 0, +W\}$. If we choose $W = 20$ to be an even multiple of J, we can expect some arguments of our discussion of fragmentation in the main text would continue to hold. In Supplementary Figure 2(g-i), we show S_E, S_N, and S_C for this model, for $W = 20$. This restores some of the phenomenology we observe in the Schwinger model, including slow growth in both S_E and S_C, and some banding in all three types of entropy.

SUPPLEMENTARY NOTE 6: SPECTRAL FORM FACTOR

Eigenstates carry information about the time $t \to \infty$ limit, which is not directly accessible to numerics otherwise. To probe the behavior of the system at finite times, we consider the Spectral Form Factor (SFF) [4, 5], defined as the Fourier transform of a two-point spectral correlation function. Crucially, the SFF quantifies *long-ranged* correlations between energy eigenvalues and thus holds more information than, e.g., the level spacing ratio r, which is only dependent on short-ranged spectral correlations. Calculating the SFF requires "unfolding" the spectrum to make the density of eigenvalues equal to unity [6], after which it is defined as

$$K(\tau) = \frac{1}{Z}\left\langle \left| \sum_{j=1}^{\mathcal{N}} e^{-2\pi i \epsilon_j \tau} \right|^2 \right\rangle , \tag{S17}$$

where $\{\epsilon_1, ..., \epsilon_D\}$ are the unfolded eigenvalues, τ is the rescaled time following the unfolding, and Z is a normalization factor chosen such that $K(\tau) \to 1$ as $\tau \to \infty$. In these units, the Heisenberg time, which is given by the inverse of the mean level spacing, is $\tau_{\mathrm{H}} = 1$. τ_{H} measures when discrete energy levels can be resolved, after which the finite nature of the system becomes evident. We furthermore choose to calculate the connected SFF, $K_c(\tau)$, which additionally subtracts a non-universal disconnected part,

$$K_c(\tau) = K(\tau) - A\left| \left\langle \sum_{j=1}^{\mathcal{N}} e^{-2\pi i \epsilon_j \tau} \right\rangle \right|^2 , \tag{S18}$$

with A again a normalization factor chosen to ensure $K_c(\tau) \to 0$ as $\tau \to 0$. This avoids the need to apply a Gaussian filter to the unfolded spectrum [6].

The SFF, in principle, provides detailed information about the distribution of eigenvalues in the system; however, for our purposes, we are mostly interested in determining whether the system exhibits chaotic dynamics. This can be done by comparing $K_c(\tau)$ to the GOE prediction, given by $K_{c,\,\mathrm{GOE}}(\tau) = \tau(2 - \ln(1 + 2\tau))$ for $\tau < 1$. In chaotic systems, $K_c(\tau)$ will agree with the GOE prediction after the (rescaled) Thouless time τ_{Th}, which can be interepreted as the time after which the dynamics are universal and the system is indistinguishable from a random matrix.

In Supplementary Figure 3(a) we show the connected SFF for several values of J and $N = 16$, alongside the GOE prediction. For small but finite $J = 0.1$, the SFF rapidly approaches the random matrix form, and therefore we can classify the system as chaotic in this regime. However, as we increase J, we see that the SFF deviates from the GOE prediction, and by $J = 2$ no longer intercepts it before $\tau_{\mathrm{H}} = 1$, but instead approaches the constant value $K_c(\tau) = 1$ well before τ_{H}; this is typical of the MBL regime [6]. The SFF takes on a particularly unusual form for $J = 5$, with plateaus observable at $\tau = (2J/w)^{-2}$ and $(2J/w)^{-3}$: these likely follow from the spectral splitting observed in the main text, and is further evidence that the system may not follow the conventional MBL phenomenology in the strong-coupling regime. While Ref. [7] has already studied the SFF of the Schwinger model, the anomalous SFF features for large J have not been pointed out.

To further quantify the crossover from chaotic to MBL dynamics, we calculate $g(N, J) = \log_{10}(\tau_{\mathrm{H}}/\tau_{\mathrm{GOE}})$ in

Supplementary Figure 3(b), where τ_{GOE} is the last time at which $K_c(\tau)$ deviates significantly from the GOE prediction, i.e., $|\log_{10}(K_c(\tau)/K_{c,\text{GOE}})| < 0.08$ for all $\tau > \tau_{\text{GOE}}$. For chaotic systems, is expected that $g \to +\infty$ in the thermodynamic limit, indicating a rapid approach to random matrix dynamics; on the other hand, $g \to -\infty$ for non-ergodic systems, indicating that they do not obey the GOE at any time before finite size effects dominate. However, in practice, $\tau_{\text{GOE}} > \tau_H$ ($g < 0$) is not physically meaningful, as beyond the Heisenberg time the discrete nature of the system is resolved and the dynamics cannot be related to that of the thermodynamic limit. The procedure for computing τ_{GOE} leads to an unknown constant offset in $g(N, J)$, but we can identify the crossover as the region in which $g \to 0^+$, which here is $J_c \approx 1$: this is shown more closely in the inset of Supplementary Figure 3(b).

SUPPLEMENTARY NOTE 7: MANY-BODY THOULESS PARAMETER

The change in the nature of entanglement as a function of coupling ratio J/w in the Schwinger model also has consequences for the response of eigenstates to local perturbations. In the ergodic phase, a local perturbation strongly hybridizes an extensive number of eigenstates. It was argued in Ref. [8] that such perturbations should only hybridize an intensive number of eigenstates in an MBL phase, as a local perturbation can only affect the degrees of freedom within a fixed localization radius of its support. For a local perturbation $\hat{V}$, the extent of the such hybridization can be probed using a dimensionless parameter – a many-body generalization of the Thouless conductance:

$$\mathcal{G}(\epsilon, N) = \ln \frac{|V_{n,n'}|}{E'_{n+1} - E'_n}, \tag{S19}$$

where $\epsilon = E'_n/N$ are the sorted energy densities of the perturbed Hamiltonian, and $V_{n,n'}$ are the matrix elements of the perturbation operator in the unperturbed eigenbasis.

In an ergodic phase, a local operator strongly mixes neighboring eigenstates, leading to $\mathcal{G} \sim \mathcal{O}(N)$. In the MBL phase, eigenstates with neighboring energies are coupled exponentially weakly, leading to $\mathcal{G} \sim -\mathcal{O}(N)$. Then, the ergodic-MBL transition point can be identified as the J where $\mathcal{G}(N) \sim \mathcal{O}(1)$. In Supplementary Figure 4, we plot the averaged Thouless parameter as a function of system size for different values of J. The averaging is over 33% of eigenstates around the DOS peak, after which we further average over charge sectors. The results are shown for a local perturbation $\hat{\sigma}^z$ on the first site of the chain with a strength $w/10$, although the results are similar for different sites and using other perturbations such as $\hat{\sigma}_i^+\hat{\sigma}_{i+1}^-$. For small J, we observe an

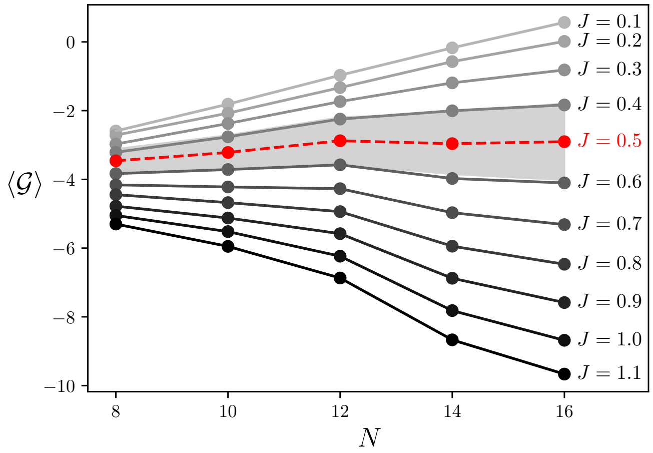

Supplementary Figure 4. The Thouless parameter $\mathcal{G}$, averaged over charge sectors, as a function of system size for different values of J and fixed $w = 1$. The averaging is done across 500 charge sectors for $N < 16$ and 450 charge sectors for $N = 16$. The perturbation is a local $\hat{\sigma}^z$ operator on the first site with strength $w/10$. The Thouless parameter is seen to become independent of system size around $J \approx 0.5$ (red dashed line). The shaded region represents the uncertainty (not to scale) in J around the transition point.

approximately linear increase of the Thouless parameter, consistent with an ergodic phase. Upon increasing J, the dependence on system size changes sign, with a decay becoming visible for $J > 0.6$, consistent with an MBL regime. We identify the region around $J \approx 0.5$ as the regime where the Thouless parameter becomes independent of system size, i.e., the ergodic-MBL transition.

SUPPLEMENTARY NOTE 8: COULOMB INTERACTIONS VERSUS DISORDER

In this section, we analyse the impact of the Coulomb interactions and disorder on the growth of entropy by tuning their magnitudes independently in the Hamiltonian. We write the full Hamiltonian as $\hat{H}_{\{q_\alpha\}} = \hat{H}_\pm + J_{ZZ}\hat{H}_{ZZ} + J_q\hat{H}_q$, where $\hat{H}_{ZZ}$ and $\hat{H}_q$ are the Coulomb and disorder terms with strengths J_{ZZ} and J_q respectively. In Supplementary Figure 5(a)-(b), we plot the number and configurational entropies starting from the $|\text{vac}\rangle$ initial state by first fixing a small disorder strength $J_q = 0.1$, and gradually increasing the Coulomb interaction strength J_{ZZ}. Both the number and configurational entropies grow roughly logarithmically, followed by a saturation value that decreases with increasing Coulomb interaction strength. Although the growth of the configurational entropy is slower, it still saturates by $wt \approx 10^2$ even for $J_{ZZ} = 10$. This implies that although the growth and the corresponding saturation value of entanglement entropy is suppressed upon increasing the Coulomb interaction strength, the Coulomb interactions are not them-

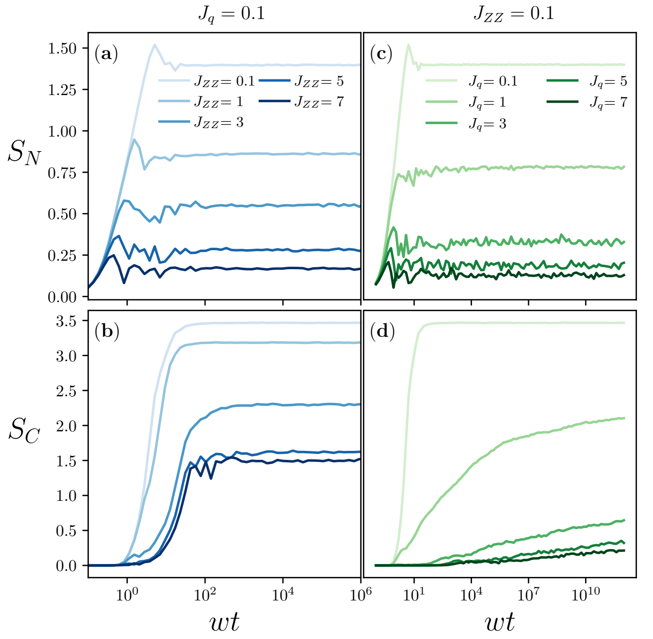

Supplementary Figure 5. (a)-(b): Growth of the number and configurational entropy with a fixed disorder strength $J_q = 0.1$ and increasing Coulomb interaction strength J_{ZZ}. The entropy growth is logarithmic when disorder is small and Coulomb interactions dominate. (c)-(d): Growth of number and configurational entropy with a fixed Coulomb interaction strength $J_{ZZ} = 0.1$ and increasing disorder strength J_q. Ultraslow growth of the configurational entropy is seen when Coulomb interactions are small and disorder dominates. All data is for the $|\text{vac}\rangle$ initial state with $N = 16$, averaged over 25 charge sectors.

selves responsible for the ultraslow growth of entropy observed at large J.

The origin of the slow growth of entanglement entropy is instead attributed to the disorder term which is controlled by J_q. In Supplementary Figure 5(c)-(d), we test this by setting the Coulomb interaction strength to a small value $J_{ZZ} = 0.1$ and gradually increasing the disorder strength J_q. Upon increasing J_q, the growth of number entropy is strongly suppressed, although it still saturates at early times. The slow growth of configurational entropy starts to emerge at around $J_q = 3$, similar to that observed in the main-text. We note that both the terms J_q and J_{ZZ} induce ergodicity-breaking individually, and the level spacing ratio varies smoothly from Wigner-Dyson to Poisson and dips further below the Poisson value as the strength of either term is increased whilst keeping the other fixed. However the nature of ergodicity-breaking induced by both terms is distinct, as only the disordered term leads to an ultraslow growth of entanglement. The role of the discrete disorder term J_q is studied in more detail in Supplementary Note 5.

SUPPLEMENTARY METHODS: DEGENERATE PERTURBATION THEORY FOR THE LATTICE SCHWINGER MODEL

In the main text we introduced the degenerate towers $\{\mathcal{K}_a\}$, with $\mathcal{K}_0$ denoting the tower containing our initial state, each with an unperturbed energy E_a. We can also define $\{\mathcal{K}_a^b\}$ as the set of Krylov subspaces within $\mathcal{K}_a$, which are generated by $\hat{H}^\pm$ projected into $\mathcal{K}_a$, and likewise let $\mathcal{K}_0^0$ contain our initial state. Let $\hat{P}_a$ and $\hat{P}_a^b$ be the projectors onto these towers and subspaces, respectively. We then define off-diagonal blocks,

$$\hat{T}_{i;j} = \hat{P}_j \hat{H}_\pm \hat{P}_i^\dagger \ . \tag{S20}$$

Note that $\hat{T}_{i;j}^\dagger = \hat{T}_{j;i}$. We can then write down,

$$\hat{H}^{[0]} = \hat{P}_0 \left(\hat{H}_{ZZ} + \hat{H}_q \right) \hat{P}_0^\dagger \ , \tag{S21}$$

$$\hat{H}^{[1]} = \hat{P}_0 \hat{H}_\pm \hat{P}_0^\dagger = \hat{T}_{0;0} \ , \tag{S22}$$

representing the full Hamiltonian projected onto the tower $\mathcal{K}_0$. Higher-order corrections may then be calculated through successive Schrieffer-Wolff transformations [9], giving the effects of virtual hops to other energy levels. The second- and third-order contributions are given by:

$$\hat{H}^{[2]} = \sum_{a \neq 0} \frac{\hat{T}_{0;a} \hat{T}_{a;0}}{E_a - E_0} \ , \tag{S23}$$

$$\begin{aligned} \hat{H}^{[3]} =& \sum_{a\neq 0} \frac{2\hat{T}_{0;a}\hat{T}_{a;a}\hat{T}_{a;0} - (\hat{T}_{0;0}\hat{T}_{0;a}\hat{T}_{a;0} + \text{H.c.})}{2(E_a - E_0)^2} \\ &+ \sum_{a \neq b} \Delta_{ab}^0 \hat{T}_{0;b} \hat{T}_{b;a} \hat{T}_{a;0} \ , \end{aligned} \tag{S24}$$

$$\Delta_{ab}^0 = \frac{(E_b - E_a) + (E_b - E_0)}{(E_a - E_0)(E_b - E_0)(E_b - E_a)} + (a \leftrightarrow b) \ . \tag{S25}$$

$\hat{H}^{[2]}$ tends to be diagonal, generating dephasing between states within a Krylov subspace, while $\hat{H}^{[3]}$ and higher generally also act off-diagonally to connect different Krylov subspaces at the same energy.

SUPPLEMENTARY REFERENCES

[1] J. Kogut and L. Susskind, Hamiltonian formulation of Wilson's lattice gauge theories, Phys. Rev. D **11**, 395 (1975).
[2] T. Banks, L. Susskind, and J. Kogut, Strong-coupling calculations of lattice gauge theories: (1 + 1)-dimensional exercises, Phys. Rev. D **13**, 1043 (1976).
[3] M. Brenes, M. Dalmonte, M. Heyl, and A. Scardicchio, Many-Body Localization Dynamics from Gauge Invariance, Phys. Rev. Lett. **120**, 030601 (2018).
[4] F. Haake, *Quantum signatures of chaos* (Springer, 1991).
[5] M. C. Gutzwiller, *Chaos in classical and quantum mechanics*, Vol. 1 (Springer Science & Business Media, 2013).

[6] J. Šuntajs, J. Bonča, T. Prosen, and L. Vidmar, Quantum chaos challenges many-body localization, Phys. Rev. E **102**, 062144 (2020).

[7] G. Giudici, F. M. Surace, J. E. Ebot, A. Scardicchio, and M. Dalmonte, Breakdown of ergodicity in disordered $U(1)$ lattice gauge theories, Phys. Rev. Res. **2**, 032034 (2020).

[8] M. Serbyn, Z. Papić, and D. A. Abanin, Criterion for Many-Body Localization-Delocalization Phase Transition, Phys. Rev. X **5**, 041047 (2015).

[9] S. Bravyi, D. P. DiVincenzo, and D. Loss, Schrieffer-Wolff transformation for quantum many-body systems, Ann. Phys. **326**, 2793 (2011).
